# Changes in lung function in children after pneumonia: a multicenter study

**DOI:** 10.1186/s13052-025-02092-7

**Published:** 2025-08-15

**Authors:** Hejun Jiang, Jingsheng Dai, Guifang Zhou, Guijun Yang, Liwen Zhang, Shuhua Yuan, Jing Zhang, Jiande Chen, Mingyu Tang, Jilei Lin, Li Li, Yufen Wu, Yong Yin

**Affiliations:** 1 Department of Respiratory Medicine, Shanghai Children’s Medical Center, Shanghai Jiao Tong University School of Medicine, Shanghai, China; 2 Department of Pediatric, Shanghai Pudong New Area People’s Hospital, Shanghai, China; 3 Department of Respiratory Medicine, Linyi Branch of Shanghai Children’s Medical Center, Linyi Maternal and Child Healthcare Hospital, Linyi City, Shandong Province China; 4 Department of Pediatric, School of Medicine, Putuo People’s Hospital, Tongji University, Shanghai, China; 5 Shanghai Children’s Medical Center Outpatient and Emergency Office, Shanghai Jiao Tong University School of Medicine, Shanghai, China; 6 Pediatric AI Clinical Application and Research Center, Shanghai Children’s Medical Center, Shanghai, China; 7 Shanghai Engineering Research Center of Intelligence Pediatrics (SERCIP), Shanghai, China; 8 Child Health Advocacy Institute, China Hospital Development Institute, Shanghai Jiao Tong University, Shanghai, China

**Keywords:** Rhinovirus, Lung function, Pneumonia, Small airway disorder, Large airway disorder

## Abstract

**Background:**

There are few studies on the changes in lung function after pneumonia in children. This study aims to explore the changes in lung function in children after pneumonia and analyze the risk factors for airway disorder, especially the impact of different pathogen infection on lung function.

**Methods:**

This study collected data from patients who were hospitalized due to pneumonia in ten Chinese hospitals between January 2023 and December 2024. Pulmonary function tests were performed to assess changes in lung function one week and one month after discharge.

**Results:**

A total of 566 children were included in this study, with 40.6% of patients still showing airway disorder one week after discharge. Different pathogenic infections had varying effects on pulmonary function. MP (Mycoplasma pneumoniae) infection [OR (95%CI): 1.881(1.268–2.789), *P* = 0.001] and RhV (rhinovirus) infection [OR (95%CI): 2.402(1.027–5.621), *P* = 0.043] were significant risk factors for the occurrence of SAD (Small Airway Disorder) one week after discharge. Male gender [OR (95%CI): 2.219, *P* = 0.001] and MP infection [OR (95%CI): 1.681(1.024–2.761), *P* = 0.039] were significant risk factors for the occurrence of LAD (Large Airway Disorder) one week after discharge. No positive pathogen results [OR (95%CI): 0.366(0.168–0.800), *P* = 0.011] were significant protective factors for the persistence of SAD one month after discharge, while RhV infection [OR (95%CI): 7.286(0.802, 66.238), *P* = 0.077] and lung consolidation [OR (95%CI): 1.753(0.956, 3.214), *P* = 0.069] showed mild significance for the persistence of SAD one month after discharge. Male gender [OR (95%CI): 2.246(1.137–4.436), *P* = 0.019] and RhV infection [OR (95%CI): 1.967(1.630–237.549), *P* = 0.019] were significant risk factors for the persistence of LAD one month after discharge, while no positive pathogen results [OR (95%CI): 0.249(0.092–0.678), *P* = 0.006] were a significant protective factor.

**Conclusions:**

Approximately 40.6% of children after pneumonia still had airway disorder one week after discharge, which was closely related to different pathogenic infections. Patients with RhV pneumonia, in particular, should be closely monitored for changes in lung function after discharge.

**Supplementary Information:**

The online version contains supplementary material available at 10.1186/s13052-025-02092-7.

## Introduction

Pneumonia is the most common disease in children worldwide and the leading cause of death in children under five years old. The number of children hospitalized due to pneumonia in 2015 increased by 187% compared to 2000 [1], indicating a rise in severe cases. Given that children's lungs are not fully developed, pneumonia can interfere with the lung development process, potentially affecting the structure and function of lung tissue during development [2, 3]. Although most cases of pneumonia in children are treatable, it is currently unclear whether pulmonary dysfunction persists after pneumonia and whether changes in pulmonary function are associated with factors such as age at illness, gender, infectious pathogens, and severity of disease.

At the same time, there is growing interest in the determinants of pulmonary function, including in early childhood pneumonia. Children's lung function is significantly associated with lung function in adulthood, as well as the future burden of cardiovascular and pulmonary diseases and all-cause mortality risk [4–8]. There is also evidence suggesting that lung growth and airway caliber improvement are possible during childhood [4, 5]. Therefore, strategies to optimize lung function in children are crucial. Given the enormous global burden of early respiratory infections and pneumonia in children, as well as the established importance of childhood pulmonary function for lung health in adulthood and future cardiovascular and pulmonary outcomes, understanding the impact of pneumonia on lung development and function during childhood is of utmost importance.

This study focuses on children hospitalized for pneumonia, exploring changes in lung function and associated factors.

## Methods

### Inclusion and exclusion criteria

**Fig.1:**
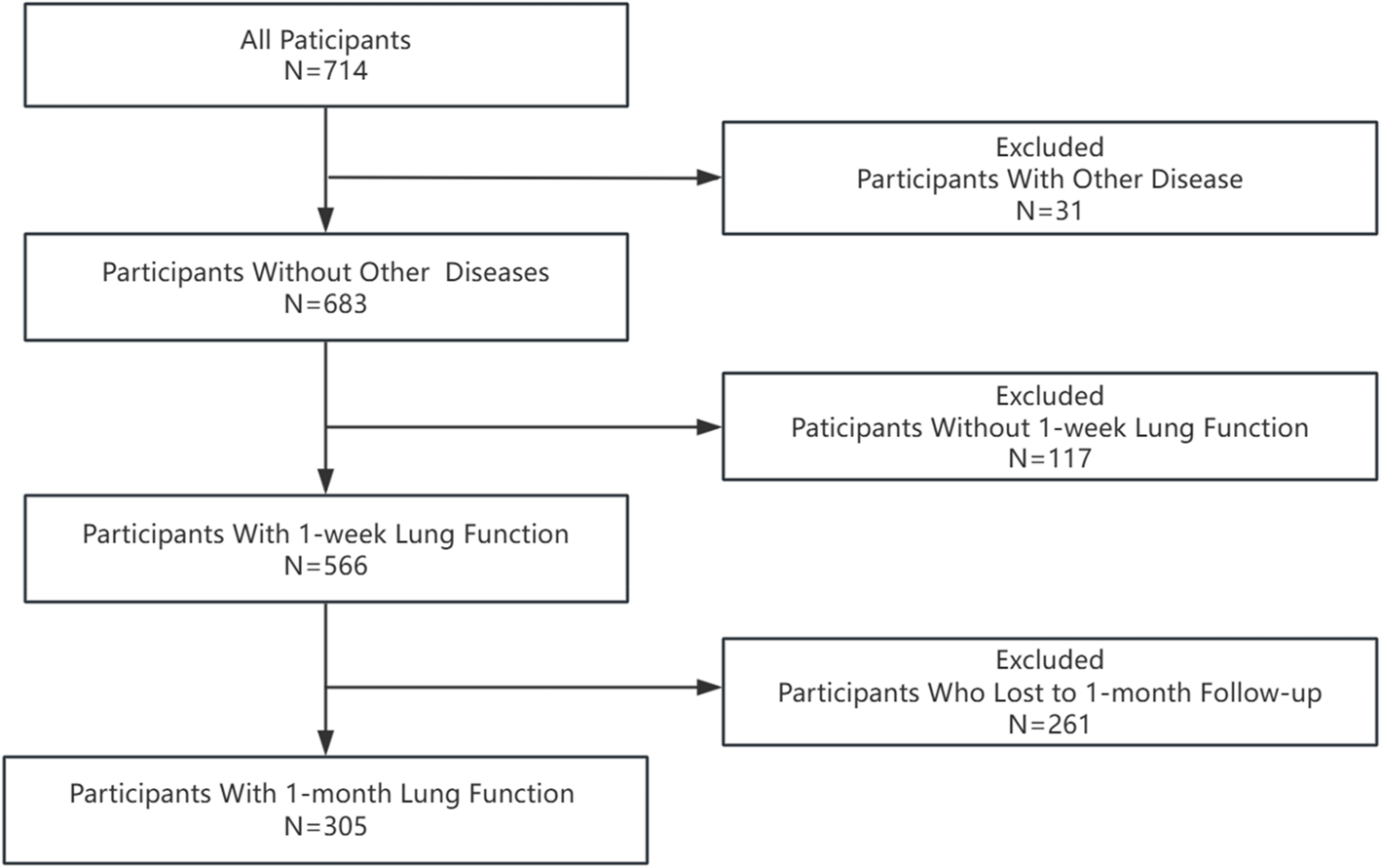
Flowchart of the research

This study collected data from patients who were hospitalized with a primary diagnosis of pneumonia at ten hospitals, including Shanghai Children's Medical Center, Shanghai Putuo People's Hospital, Shanghai Pudong New Area People's Hospital, and Linyi Maternal and Child Healthcare Hospital, between January 2023 and December 2024, and who attended outpatient follow-up visits one week and one month after discharge. Exclusion criteria were: 1) Acute respiratory infection symptoms persisting one week after discharge; 2) Respiratory infection recurrence one month after discharge; 3) Comorbidities such as asthma, bronchiectasis, congenital airway and lung developmental abnormalities, interstitial lung disease, etc.; 4) Refusal to participate in the study. The study was approved by the Medical Ethics Committee of Shanghai Children's Medical Center, affiliated with Shanghai Jiao Tong University (Approval No.: SCMCIRB-K2024126-1), and informed consent was obtained from the guardians of the children. The detailed procedure is shown in Fig. 1.

### Data collection

According to the guidelines of the European Respiratory Society/American Thoracic Society [9], children aged 3–6 years underwent impulse oscillometry (IOS), while children older than 6 years underwent lung ventilation function tests. Forced vital capacity (FVC), forced expiratory volume in 1 s (FEV1), and peak expiratory flow (PEF) ≥ 80% of the predicted value were considered normal. An FEV1/FVC ratio > 80% was also considered normal. Mid-expiratory flow (MMEF), forced expiratory flow at 50% of vital capacity (FEF50), and forced expiratory flow at 75% of vital capacity (FEF75) ≥ 65% of the predicted value were considered normal. An FEV1/FVC ratio < 0.80 was defined as large airway disorder (LAD). When two or more of FEF25, FEF50, and FEF75 were below 65% of the predicted value, the patient was considered to have small airway dysfunction (SAD). Patients with LAD and/or SAD were defined as having airway disorder (AD). Lung function measured one week after discharge was used as the baseline pulmonary function. In this study, the pulmonary function values presented were ratios of the measured values to the predicted values.

### Covariates

Pneumonia and severe pneumonia were diagnosed according to the Chinese Pediatric Community-Acquired Pneumonia Guidelines (2024) [10]. Lung consolidation, pleural effusion, and other conditions were defined using chest X-rays and/or chest CT scans taken during hospitalization. Microbiological testing was conducted during hospitalization for pathogen detection, comprising of respiratory multiplex PCR assays targeting 22 common respiratory pathogens from throat swabs, sputum and blood cultures, serological testing and BALF-based pathogen testing (when available). If the patient had a positive PCR result for the pathogen in throat swabs or positive sputum culture during hospitalization, along with corresponding symptoms of acute respiratory infection, the patient was considered to be infected with that pathogen. Patients were classified as having no positive pathogen results if all available microbiological tests returned negative results. If the patient had no hypoxemia, severe pneumonia, lung consolidation, pleural effusion, or atelectasis during hospitalization for respiratory infection, the patient was considered to have no pulmonary complications.

### Data analysis

All data analyses were performed using R version 4.3.2. A two-sample t-test was used to compare differences in pulmonary function parameters between positive and negative pathogen groups. Univariate linear regression was used to assess the correlation between independent variables and continuous outcome variables, while univariate logistic regression was used to evaluate the correlation between independent variables and categorical outcome variables. Considering the potential multicollinearity between independent variables, Lasso regression was used to identify variables affecting the occurrence of LAD and SAD one week after discharge, as well as one month after discharge. The candidate variables included demographic factors (age, sex), pathogen factors, pulmonary complications, clinical severity (whether the case was classified as severe community-acquired pneumonia, SCAP), length of hospital stay, and the types of antibiotics administered during hospitalization. Based on the variables selected through LASSO regression, the final multivariate logistic regression models were established.

## Results

### Baseline

Table 1 shows the baseline characteristics of all participants. A total of 566 patients were included in this study. In this study, at baseline pulmonary function one week after discharge, 230 out of 566 patients (40.6%) exhibited airway disorder. Among these patients, the proportion of MP infection was higher, as was the proportion of RhV infection. Additionally, the rate of lung consolidation during hospitalization was significantly higher, and these patients often had positive microbiological test results. No significant differences were observed between groups for the remaining variables. A total of 314 patients had pulmonary function measurements one month after discharge. Among the 230 patients with airway disorder one week after discharge, 107 did not have lung function measurements one month later. Of the 230 patients with SAD one week after discharge, 123 attended the follow-up one month later. Among these patients, 73 (59.3%) recovered from SAD. In addition, patients with airway dysfunction at one month had significantly longer hospital stays, while there were no significant differences in the classes of antibiotics used between groups (Supplementary Material).

**Table 1:**
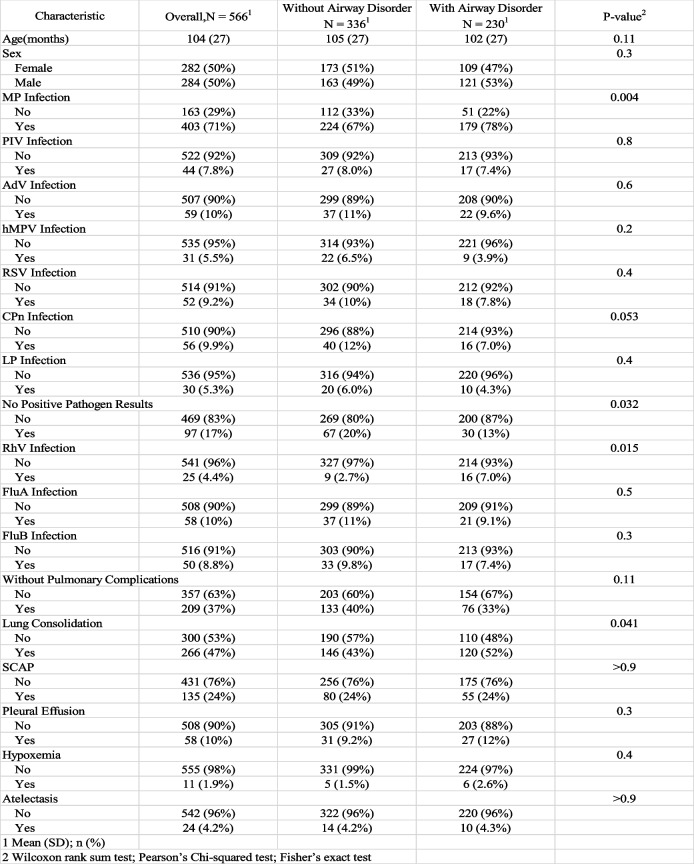
Baseline of the study

*MP Mycoplasma pneumoniae *PIV* Parainfluenza virus, *Adv* Adenovirus, *hMPV* Human metapneumovirus, *RSV* Respiratory syncytial virus, *CpN* Chlamydia pneumoniae, *LP* Legionella pneumophila, *RhV* Rhinovirus, *FluA* Influenza A virus, *FluB* Influenza B virus, *SCAP* Severe community-acquired pneumonia

### Baseline lung function and different pathogen infections

**Fig. 2:**
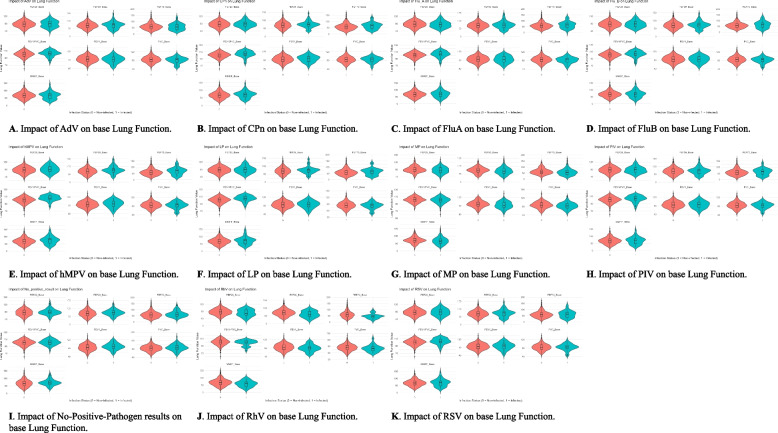
Violin plots showing the impact of different pathogens on various baseline lung function parameters

Figure 2 shows the pulmonary function parameters for patients with positive and negative pathogen results one week after discharge. One week after discharge, patients with MP infection had lower FVC (Δ = −3.558, *P* = 0.024), FEV1 (Δ = −4.315, *P* = 0.006), FEF25 (Δ = −4.497, *P* = 0.013), FEF50 (Δ = −5.122, *P* = 0.013), FEF75 (Δ = −5.096, *P* = 0.049), and MMEF (Δ = −5.658, *P* = 0.011) compared to other patients. Patients with hMPV (Δ = 5.471, *P* = 0.001), CPn (Δ = 3.299, *P* = 0.014), and LP (Δ = 4.581, *P* = 0.005) infections had higher FEV1/FVC compared to other patients. Patients with no positive pathogen results had higher FEV1 (Δ = 4.072, *P* = 0.023), FEF50 (Δ = 4.613, *P* = 0.042), FEF75 (Δ = 6.129, *P* = 0.035), and MMEF (Δ = 5.484, *P* = 0.025) compared to other patients. Patients with RhV infection had lower FEF50 (Δ = −9.767, *P* = 0.028), FEF75 (Δ = −13.061, *P* = 0.005), and MMEF (Δ = −9.084, *P* = 0.043) compared to other patients. Patients with FluA infection had higher FEV1/FVC ratios (Δ = 2.723, *P* = 0.043) compared to other patients. No significant differences were observed for the remaining variables. Detailed data can be found in the Supplementary Materials.

### Lung function one month after discharge and different pathogen infections

**Fig. 3:**
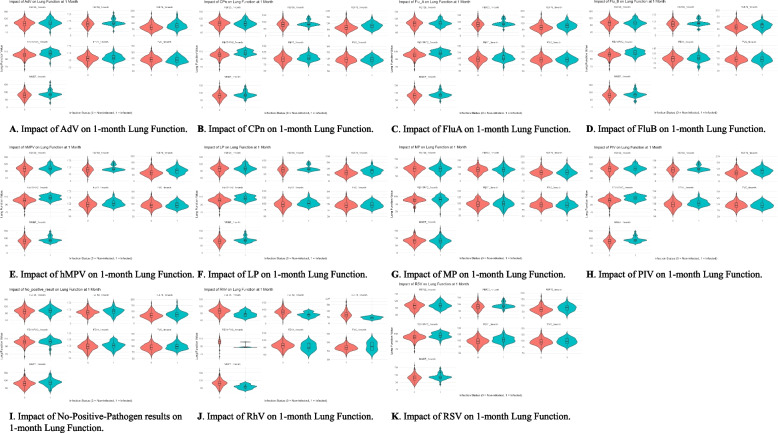
Violin plots showing the impact of different pathogens on various lung function parameters one month after discharge

Figure 3 shows the pulmonary function one month after discharge for patients with positive and negative pathogen results. Patients with RhV infection had significantly lower FEV1/FVC (Δ = −10.959, *P* = 0.038), FEF75 (Δ = −25.276, *P* = 0.003), and MMEF (Δ = −22.806, *P* = 0.020) compared to other patients. Children with no positive pathogen results had higher FVC (Δ = 3.598, *P* = 0.038), FEV1 (Δ = 4.584, *P* = 0.019), and MMEF (Δ = 6.692, *P* = 0.036) compared to other children. Patients with PIV, AdV, hMPV, RSV, CPn, LP, FluA, and FluB infections also showed significant differences in pulmonary function one month after discharge compared to other patients. To further investigate whether heterogeneity exists within the RhV infection group due to co-infection with other pathogens, we performed a subgroup analysis comparing children with single RhV infection and those with co-infections. The results showed no significant differences in pulmonary function parameters or the incidence of airway dysfunction at both baseline and 1 month after discharge. Detailed results can be found in the Supplementary Materials.

### Univariate regression of independent variables and lung function parameters

**Fig. 4:**
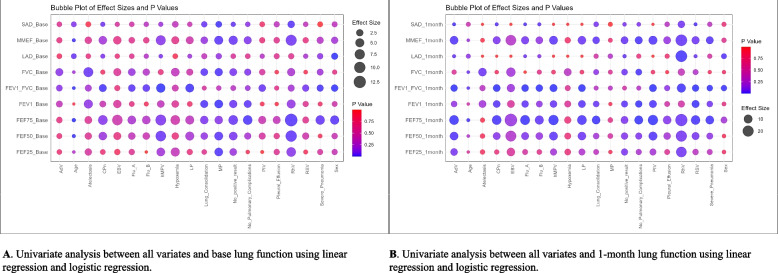
Univariate regression of independent variables and lung function parameters

Figure 4 shows the results of univariate regression of independent variables and lung function parameters. RhV infection was a risk factor for the occurrence of SAD one week after discharge (OR = 2.716, *P* = 0.016) and was significantly negatively correlated with FEF75 (β = −12.934, *P* = 0.016) and FEF50 (β = −9.862, *P* = 0.026). At the same time, no positive pathogen results was a protective factor for the occurrence of SAD one week after discharge (OR = 0.602, *P* = 0.033) and was significantly positively correlated with FEF75 (β = 6.137, *P* = 0.036), MMEF (β = 5.323, *P* = 0.037), and FEV1 (β = 3.712, *P* = 0.041). RhV infection was also a significant risk factor for the persistence of LAD one month after discharge (OR = 22.869, *P* = 0.006) and was significantly negatively correlated with FEV1/FVC (β = −10.959, *P* = 0.004), FEF75 (β = −25.276, *P* = 0.039), and MMEF (β = −22.806, *P* = 0.032) one month after discharge. RhV infection showed a mild significant association with the persistence of SAD one month after discharge (OR = 5.857, *P* = 0.056). No positive pathogen results remained a protective factor for the occurrence of LAD one month after discharge (OR = 0.347, *P* = 0.032) and showed a mild significant association with the occurrence of SAD (OR = 0.495, *P* = 0.069).

### Multivariable regression model for factors influencing the occurrence of SAD and LAD

**Fig. 5:**
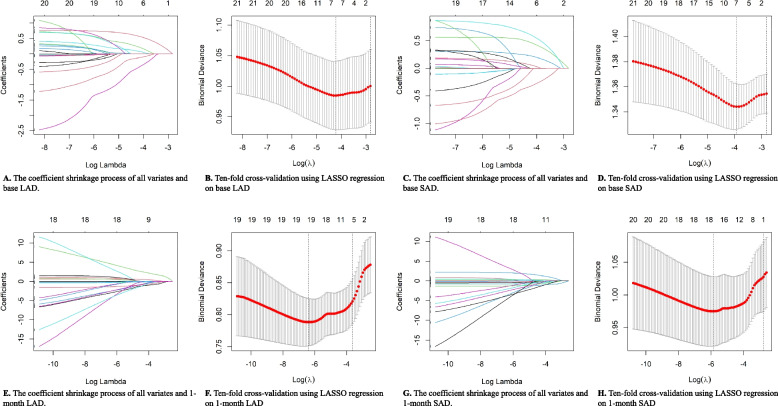
The process of establishing the final multivariable regression model using Lasso regression

We used Lasso regression to establish the final multivariable regression model, and the detailed process is shown in Fig. 5. Table 2 presents the final established multivariable regression model. We found that MP infection [OR (95%CI): 1.881 (1.268–2.789), *P* = 0.001] and RhV infection [OR (95%CI): 2.402 (1.027–5.621), *P* = 0.043] were significant risk factors for the occurrence of SAD one week after discharge. Male gender [OR (95%CI): 2.219, *P* = 0.001] and MP infection [OR (95%CI): 1.681 (1.024–2.761), *P* = 0.039] were significant risk factors for the occurrence of LAD one week after discharge. No positive pathogen results [OR (95%CI): 0.366 (0.168–0.800), *P* = 0.011] was a significant protective factor for the persistence of SAD one month after discharge, while RhV infection [OR (95%CI): 7.286 (0.802, 66.238), *P* = 0.077], lung consolidation [OR (95%CI): 1.753 (0.956, 3.214), *P* = 0.069], and AdV infection [OR (95%CI): 0.201 (0.031–1.313), *P* = 0.094] showed mild significance in the persistence of SAD one month after discharge. Male gender [OR (95%CI): 2.246 (1.137–4.436), *P* = 0.019] and RhV infection [OR (95%CI): 1.967 (1.630–237.549), *P* = 0.019] were significant risk factors for the persistence of LAD one month after discharge, while no positive pathogen results [OR (95%CI): 0.249 (0.092–0.678), *P* = 0.006] was a significant protective factor for the persistence of LAD. No significant results were observed for the remaining variables.

**Table 2:**
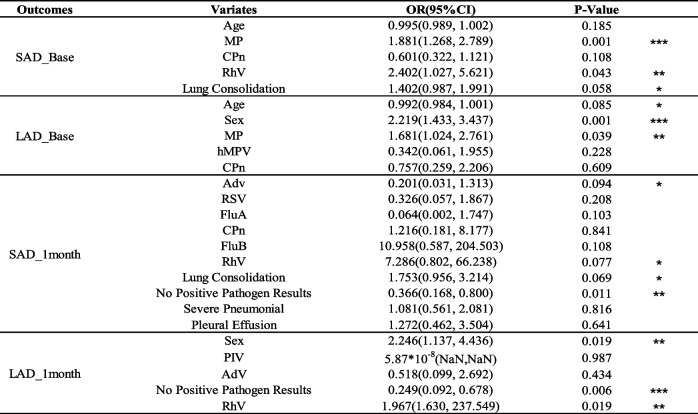
Odds ratios (OR) and *P*-values for each variable in the multivariable regression model

*** *P* ≤ 0.001; ** 0.001 < *P* < 0.05; *0.05 ≤ *P* < 0.1

## Discussion

The respiratory system in children is underdeveloped; the surface area and volume of the gas exchange portion of the lungs increase after birth, along with the maturation of alveolar structure and an increase in alveolar surface area. Animal experiments have confirmed that lung development continues into early adulthood [11, 12]. Pneumonia during childhood can interfere with or disrupt the process of lung development, potentially affecting the structure and function of lung tissue to varying degrees during development. Studies in adults have found that pneumonia during childhood is a risk factor for chronic obstructive pulmonary disease (COPD) [11], but research on the changes in lung function after pneumonia is currently lacking. Furthermore, most studies on the impact of lower respiratory tract infections (LRTIs) during early life on lung function rely on surveys, and there are few longitudinal studies on this topic. Additionally, few studies have incorporated factors such as the type of infectious pathogen, the severity of pneumonia, and chest imaging findings [13]. This study is a multicenter study that ultimately included 566 children after pneumonia, and examined the impact of different pathogens, lung imaging findings, and the severity of pneumonia on post-pneumonia lung function, which is rare in current research. However, due to limitations in objective conditions, the follow-up period in this study was only one month. Although we recommended that all patients in this study adhere to follow-up visits, only 56.88% (305/566) were available for follow-up one month after discharge. By three months post-discharge, all patients had been lost to follow-up, making it difficult to assess the long-term impact of pneumonia on lung function.

Previous studies have shown that the decline in lung function can persist from childhood into adulthood, and this decline is often manifested as a decrease in FEV1 and FVC [4–8, 14]. However, reports on changes in small airway function after pneumonia are relatively scarce [15]. In this study, among the 123 SAD patients with complete lung function data one month after discharge, only 59.3% (73/123) showed recovery of SAD. Especially for patients with RhV infection, monitoring post-discharge lung function is particularly important.

Rhinovirus is one of the important pathogens of respiratory infections in children [16], and is closely associated with acute wheezing, bronchiolitis, and exacerbation of asthma in children [17, 18]. In previous studies, van der Zalm et al. [19] reported that airway resistance in the first two months after birth is associated with rhinovirus infection and the occurrence of wheezing in the first year of life. Furthermore, RhV-associated wheezing in early childhood is closely associated with a decline in FEV1 and FEV1/FVC between the ages of 5–8 years [20]. It is also closely related to airway hyperresponsiveness and even the development of asthma [21]. This may be due to a lower interferon (IFN) response in the respiratory tract of children, leading to more severe lung damage after lower respiratory tract RhV infection [22]. We found that RhV infection was a risk factor for the occurrence of LAD (OR = 1.967, *P* = 0.019) and SAD (OR = 7.286, *P* = 0.077) one week and one month after discharge. This may be related to inflammation mediated by cytokines (such as IL-6) [21], and such an inflammatory response may lead to necrosis and shedding of airway epithelial cells, increased mucus production in the airways, and even the obstruction of small airways and increased airway reactivity [21], ultimately leading to a decline in lung function.

We found that the absence of identifiable pathogens was associated with a reduced risk of post-pneumonia airway dysfunction. Although infection is the primary cause of community-acquired pneumonia (CAP), pathogen-negative CAP still accounts for a substantial proportion of clinical cases. For instance, in the CDC EPIC study, 19% of over 2,300 hospitalized pediatric CAP cases had no pathogen identified despite comprehensive testing including PCR, culture, and serology [23]. Similarly, in our study, 17% of children with pneumonia remained pathogen-negative, even after the application of multiple microbiological diagnostic methods. Pathogen-negative pneumonia cases may not simply be an artifact of testing limitations. Rather, they may represent a distinct clinical subgroup, possibly influenced by low pathogen burden at the time of presentation or by infections caused by currently unrecognized or untargeted pathogens. Some of these infections may be self-limiting or may respond well to empirical antimicrobial and supportive therapy without requiring pathogen-specific treatment. Although current literature has not specifically examined the long-term airway function outcomes of pathogen-negative pneumonia, existing studies have shown that severe pneumonia or infections caused by specific pathogens are more likely to lead to persistent airway dysfunction [24]. Our findings suggest that pneumonia cases with no positive pathogen results were associated with a lower risk of both small and large airway dysfunction, potentially indicating a milder disease phenotype or a different immunopathogenic mechanism. The pathogen-negative group may experience less airway tissue injury due to the absence of certain pathogen-specific virulence factors, thereby facilitating better preservation of normal pulmonary function.

Previous studies have shown that abnormal pulmonary imaging is closely associated with abnormal lung function results. Several studies on adult COPD and asthma have shown that quantitative measurements from computed tomography (CT) scans are significantly correlated with lung function test results [25, 26], and this correlation is also meaningful in emphysema cohort studies and genetic epidemiological studies of COPD [27, 28]. A retrospective study that included 23 patients with post-infectious obstructive bronchiolitis found that the cross-sectional area of the airway wall was negatively correlated with FEV1 before using bronchodilators [29]. In this study, we found that lung consolidation showed slight significance in relation to the occurrence of SAD one week (*P* = 0.058) and one month (*P* = 0.069) after discharge. We believe that this slight significance may be due to the fact that in this study, lung consolidation was defined based on chest CT and X-ray findings, without considering the location, the number of affected lung lobes, or the extent of consolidation. Further imaging annotations (e.g., the lung lobe where the consolidation is located, the proportion of the consolidation volume in that lobe, etc.) may be needed to evaluate the correlation between abnormal lung imaging during pneumonia and subsequent lung function. Meanwhile, in this study, no significant correlation was found between pulmonary imaging findings such as pleural effusion and atelectasis during pneumonia hospitalization and post-pneumonia lung function. However, because these imaging findings have a low incidence in clinical practice, the sample size for these cases was insufficient, making it difficult to clarify the true relationship between these imaging features during pneumonia and lung function.

In addition to imaging findings, we also examined the potential impact of clinical severity and treatment-related factors.

In addition to pulmonary imaging findings, clinical severity and treatment-related factors were also found to be associated with post-pneumonia lung function. A cohort study of children with necrotizing pneumonia showed that longer hospital stays and more intensive treatment were associated with delayed radiologic and functional recovery [30]. In our study, children with airway dysfunction one month after discharge had significantly longer hospital stays, which may reflect more severe illness, delayed recovery, or a suboptimal response to initial treatment.

In summary, our study provides novel insights into the impact of pneumonia on pediatric lung function. The identification of rhinovirus infection as potential risk factors for post-pneumonia airway dysfunction underscores the importance of early monitoring and follow-up of respiratory function in selected patients. Nevertheless, the limited follow-up duration and sample attrition highlight the need for future longitudinal studies to track the long-term respiratory sequelae of childhood pneumonia. These efforts are essential for informing clinical strategies to prevent or mitigate long-term pulmonary consequences in children recovering from pneumonia.

## Conclusion

Approximately 40.6% of children with pneumonia still have impaired lung function one week after discharge, which is closely related to different pathogen infections. It is recommended that children with RhV pneumonia undergo lung function testing after discharge.

## Supplementary Information

Supplementary Material 1.

## Data Availability

Data will be made available on request.
