# Supplementary material for: Changes in lung function in children after pneumonia: a multicenter study
Source: Ital J Pediatr. 2025 Aug 15;51:252. doi: 10.1186/s13052-025-02092-7 (PMC12355759; doi:10.1186/s13052-025-02092-7)
Supplement: Supplementary file 1 — Supplementary Material 1. [file 13052_2025_2092_MOESM1_ESM.docx]

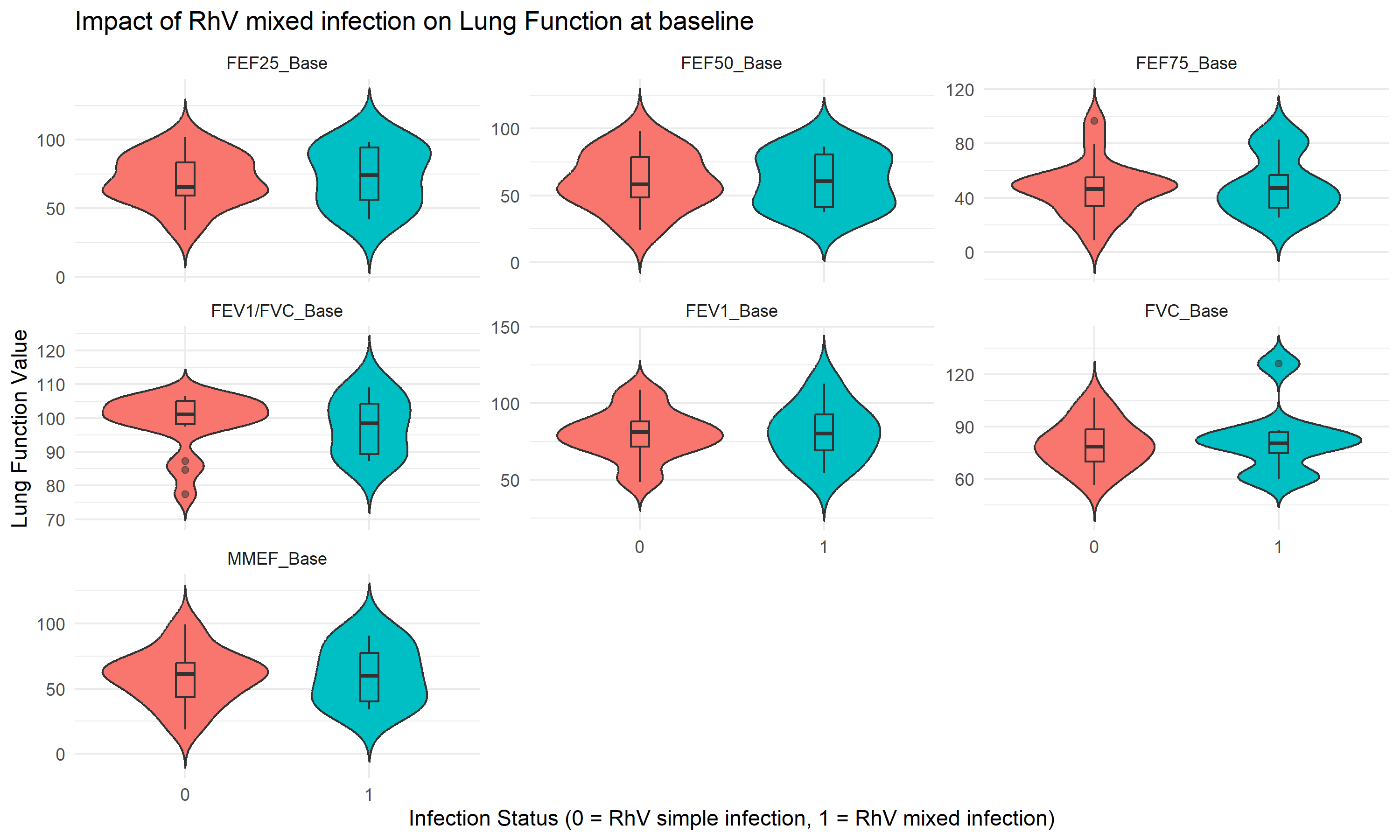


Fig 1. The impact of RhV mixed infection on various baseline lung function parameters.


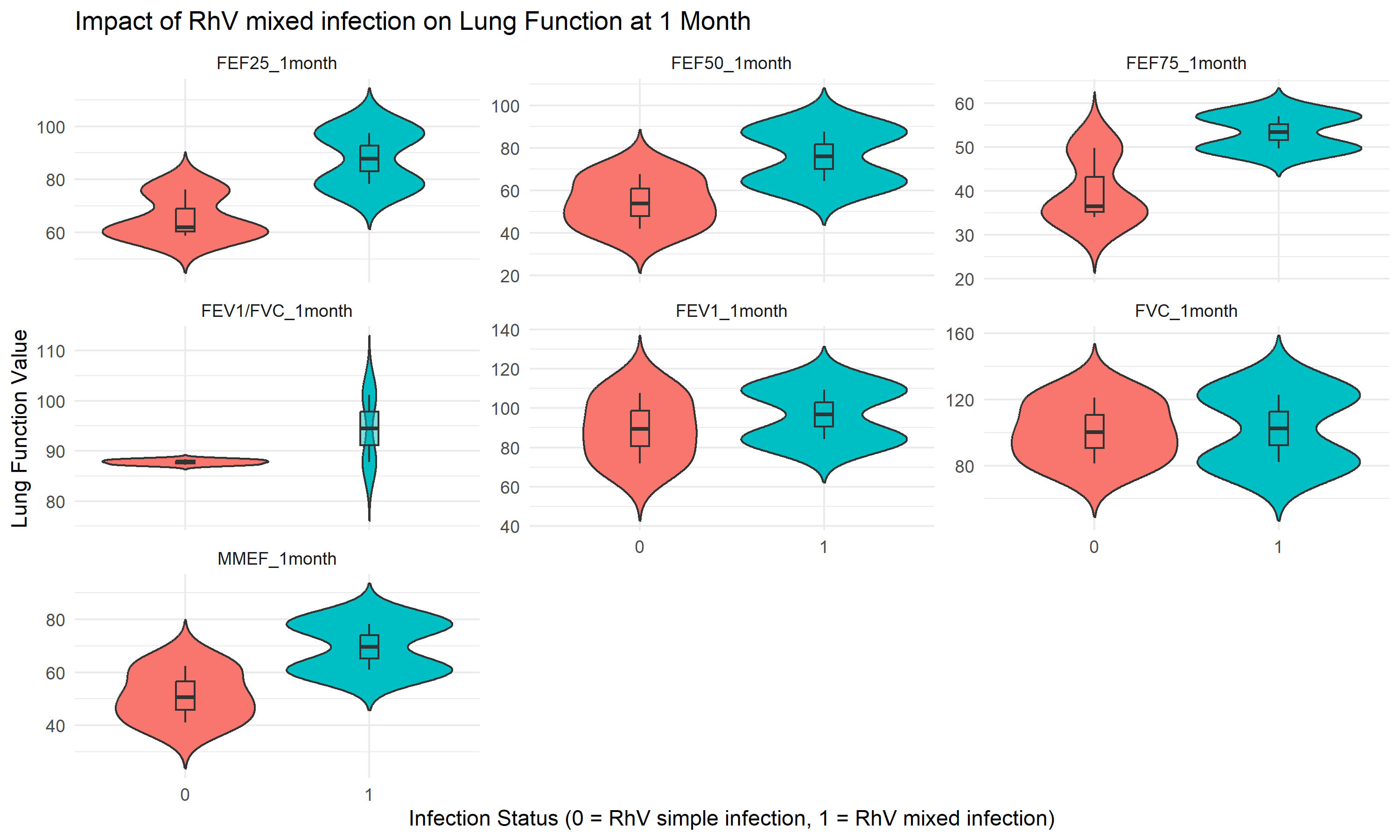


Fig 2. The impact of RhV mixed infection on various 1-month lung function parameters.

| **Lung Function** | **All RhV infection N=25** | **RhV Simple infection N=17** | **RhV mixed infection N=8** | **P value** |
| --- | --- | --- | --- | --- |
| FVC | 80.79 (15.61) | 79.83 (13.53) | 82.84 (20.23) | 0.710 |
| FEV1 | 80.08 (16.60) | 79.68 (16.11) | 80.93 (18.73) | 0.874 |
| FEV1/FVC | 98.45 (8.18) | 98.79 (8.24) | 97.71 (8.58) | 0.770 |
| FEF25 | 71.50 (19.12) | 70.75 (18.25) | 73.10 (22.10) | 0.798 |
| FEF50 | 61.01 (20.46) | 60.98 (21.08) | 61.09 (20.50) | 0.990 |
| FEF75 | 47.66 (20.61) | 46.92 (20.65) | 49.23 (21.86) | 0.806 |
| MMEF | 59.59 (20.77) | 59.07 (20.58) | 60.70 (22.54) | 0.865 |

Table 1. The impact of RhV mixed infection on various baseline lung function parameters.

| **Lung Function** | **All RhV infection N=5** | **RhV simple infection N=3** | **RhV mixed infection**  **N=2** | **P value** |
| --- | --- | --- | --- | --- |
| FVC | 101.52 (20.20) | 100.83 (20.00) | 102.55 (28.78) | 0.949 |
| FEV1 | 92.46 (16.01) | 89.60 (18.01) | 96.75 (17.75) | 0.699 |
| FEV1/FVC | 90.45 (6.02) | 87.76 (0.55) | 94.50 (9.48) | 0.498 |
| FEF25 | 74.48 (15.44) | 65.57 (9.27) | 87.85 (13.65) | 0.209 |
| FEF50 | 62.99 (17.04) | 54.39 (12.90) | 75.90 (16.55) | 0.272 |
| FEF75 | 45.39 (9.76) | 40.08 (8.46) | 53.35 (5.16) | 0.118 |
| MMEF | 58.65 (13.98) | 51.36 (10.75) | 69.60 (12.30) | 0.229 |

Table 2. The impact of RhV mixed infection on various 1-month lung function parameters.

| **Airway disorder** | **All RhV infection N=25** | **RhV simple infection N=17** | **RhV mixed infection N=8** | **P value** |
| --- | --- | --- | --- | --- |
| SAD_Base | 16 (64.0) | 11 (64.7) | 5 (62.5) | 1.000 |
| LAD_Base | 6 (24.0) | 3 (17.6) | 3 (37.5) | 0.344 |

Table 3. The impact of RhV mixed infection on airway disorders at baseline.

| **Airway disorder** | **All RhV infection N=5** | **RhV simple infection N=3** | **RhV mixed infection N=2** | **P value** |
| --- | --- | --- | --- | --- |
| SAD_1month | 3 (60.0) | 2 (66.7) | 1 (50.0) | 1.000 |
| LAD_1month | 4 (80.0) | 3 (100.0) | 1 (50.0) | 0.400 |

Table 4. The impact of RhV mixed infection on airway disorders one month after discharge.

| **Variables** | **Overall (N=566)** | **1 week after discharge** | | | **1 month after discharge** | | |
| --- | --- | --- | --- | --- | --- | --- | --- |
|  |  | **Without airway disorder (N=336)** | **With airway disorder (N=230)** | **P value** | **Without airway disorder (N=232)** | **With airway disorder (N=73)** | **P value** |
| Hospital stay (days) | 6.88 (2.67) | 6.83 (2.55) | 6.95 (2.84) | 0.617 | 6.40 (2.75) | 7.36 (3.01) | 0.012* |
| Macrolide | 340 (60.1) | 200 (59.5) | 150 (65.2) | 0.171 | 124 (53.4) | 34 (46.6) | 0.305 |
| Tetracycline | 278 (49.1) | 174 (51.8) | 104 (45.2) | 0.125 | 132 (56.9) | 36 (49.3) | 0.256 |
| Quinolone | 27 (4.8) | 19 (5.7) | 8 (3.5) | 0.233 | 10 (4.3) | 7 (9.6) | 0.086 |
| β-lactam | 2 (0.4) | 2 (0.6) | 0 (0.0) | 0.241 | 2 (0.9) | 0 (0.0) | 0.426 |
| Advanced antibiotics | 1 (0.2) | 1 (0.3) | 0 (0.0) | 0.408 | 0 (0.0) | 0 (0.0) | NA |

Table 5. Hospital stay duration and antibiotic use among patients with and without airway dysfunction at 1 week and 1 month after discharge.

| Pathogen | LungFunction | PValue | Significant | MeanDifference |
| --- | --- | --- | --- | --- |
| MP | FVC_1month | 0.557413 | No | -0.90951 |
| MP | FEV1_1month | 0.875098 | No | -0.27751 |
| MP | FEV1/FVC_1month | 0.273287 | No | 1.057376 |
| MP | FEF25_1month | 0.86443 | No | 0.409014 |
| MP | FEF50_1month | 0.495377 | No | 1.892709 |
| MP | FEF75_1month | 0.623178 | No | 1.552712 |
| MP | MMEF_1month | 0.74582 | No | 0.893507 |
| PIV | FVC_1month | 0.772853 | No | 0.595591 |
| PIV | FEV1_1month | 0.001475 | Yes | 6.922724 |
| PIV | FEV1/FVC_1month | 1.89E-07 | Yes | 6.773164 |
| PIV | FEF25_1month | 0.133874 | No | 4.510015 |
| PIV | FEF50_1month | 0.004084 | Yes | 10.36148 |
| PIV | FEF75_1month | 0.000743 | Yes | 15.44288 |
| PIV | MMEF_1month | 0.003261 | Yes | 10.51816 |
| AdV | FVC_1month | 0.513726 | No | -1.25561 |
| AdV | FEV1_1month | 0.117458 | No | 3.511609 |
| AdV | FEV1/FVC_1month | 3.82E-05 | Yes | 5.298071 |
| AdV | FEF25_1month | 0.061537 | No | 5.935977 |
| AdV | FEF50_1month | 0.01118 | Yes | 9.363407 |
| AdV | FEF75_1month | 0.005627 | Yes | 12.94532 |
| AdV | MMEF_1month | 0.014101 | Yes | 9.22116 |
| hMPV | FVC_1month | 0.743059 | No | -0.71707 |
| hMPV | FEV1_1month | 0.012961 | Yes | 5.891431 |
| hMPV | FEV1/FVC_1month | 3.25E-07 | Yes | 7.057845 |
| hMPV | FEF25_1month | 0.13351 | No | 4.84008 |
| hMPV | FEF50_1month | 0.010998 | Yes | 10.1006 |
| hMPV | FEF75_1month | 0.001975 | Yes | 15.28699 |
| hMPV | MMEF_1month | 0.009585 | Yes | 10.14656 |
| RSV | FVC_1month | 0.246658 | No | 2.376805 |
| RSV | FEV1_1month | 0.001058 | Yes | 7.131808 |
| RSV | FEV1/FVC_1month | 4.06E-05 | Yes | 5.132532 |
| RSV | FEF25_1month | 0.265038 | No | 3.253391 |
| RSV | FEF50_1month | 0.030949 | Yes | 7.058562 |
| RSV | FEF75_1month | 0.006637 | Yes | 11.9779 |
| RSV | MMEF_1month | 0.025235 | Yes | 7.641923 |
| CPn | FVC_1month | 0.818671 | No | 0.466741 |
| CPn | FEV1_1month | 0.004929 | Yes | 6.128543 |
| CPn | FEV1/FVC_1month | 4.31E-07 | Yes | 6.17102 |
| CPn | FEF25_1month | 0.663731 | No | 1.323318 |
| CPn | FEF50_1month | 0.066055 | No | 6.651587 |
| CPn | FEF75_1month | 0.004547 | Yes | 12.72291 |
| CPn | MMEF_1month | 0.042869 | Yes | 7.281463 |
| LP | FVC_1month | 0.761777 | No | 0.6788 |
| LP | FEV1_1month | 0.004889 | Yes | 6.567892 |
| LP | FEV1/FVC_1month | 3.35E-06 | Yes | 6.309799 |
| LP | FEF25_1month | 0.187114 | No | 4.342576 |
| LP | FEF50_1month | 0.019447 | Yes | 9.044749 |
| LP | FEF75_1month | 0.005329 | Yes | 13.12159 |
| LP | MMEF_1month | 0.01675 | Yes | 9.105028 |
| No_positive_result | FVC_1month | 0.038688 | Yes | 3.598881 |
| No_positive_result | FEV1_1month | 0.019032 | Yes | 4.584246 |
| No_positive_result | FEV1/FVC_1month | 0.455359 | No | 0.824935 |
| No_positive_result | FEF25_1month | 0.05803 | No | 4.948771 |
| No_positive_result | FEF50_1month | 0.093247 | No | 5.367573 |
| No_positive_result | FEF75_1month | 0.081644 | No | 6.591532 |
| No_positive_result | MMEF_1month | 0.036357 | Yes | 6.692351 |
| RhV | FVC_1month | 0.576801 | No | 5.492667 |
| RhV | FEV1_1month | 0.529378 | No | -4.94567 |
| RhV | FEV1/FVC_1month | 0.014164 | Yes | -10.9595 |
| RhV | FEF25_1month | 0.061355 | No | -17.7165 |
| RhV | FEF50_1month | 0.055492 | No | -20.2476 |
| RhV | FEF75_1month | 0.002691 | Yes | -25.2767 |
| RhV | MMEF_1month | 0.020178 | Yes | -22.8063 |
| Flu_A | FVC_1month | 0.992906 | No | -0.01827 |
| Flu_A | FEV1_1month | 0.01365 | Yes | 5.631556 |
| Flu_A | FEV1/FVC_1month | 2.17E-06 | Yes | 5.883606 |
| Flu_A | FEF25_1month | 0.420622 | No | 2.368188 |
| Flu_A | FEF50_1month | 0.047363 | Yes | 7.09169 |
| Flu_A | FEF75_1month | 0.002865 | Yes | 13.74982 |
| Flu_A | MMEF_1month | 0.028064 | Yes | 7.736535 |
| Flu_B | FVC_1month | 0.630223 | No | -1.11509 |
| Flu_B | FEV1_1month | 0.118496 | No | 4.102673 |
| Flu_B | FEV1/FVC_1month | 6.9E-05 | Yes | 5.457998 |
| Flu_B | FEF25_1month | 0.578299 | No | 1.80894 |
| Flu_B | FEF50_1month | 0.119174 | No | 6.161956 |
| Flu_B | FEF75_1month | 0.026315 | Yes | 10.60024 |
| Flu_B | MMEF_1month | 0.11298 | No | 6.260753 |
| MP | FVC_Base | 0.024447 | Yes | -3.55837 |
| MP | FEV1_Base | 0.006908 | Yes | -4.31595 |
| MP | FEV1/FVC_Base | 0.26239 | No | -0.92535 |
| MP | FEF25_Base | 0.013248 | Yes | -4.49741 |
| MP | FEF50_Base | 0.013437 | Yes | -5.1224 |
| MP | FEF75_Base | 0.049898 | Yes | -5.09651 |
| MP | MMEF_Base | 0.011348 | Yes | -5.65831 |
| PIV | FVC_Base | 0.555764 | No | -1.35448 |
| PIV | FEV1_Base | 0.81353 | No | 0.622366 |
| PIV | FEV1/FVC_Base | 0.053421 | No | 2.821123 |
| PIV | FEF25_Base | 0.970268 | No | 0.103843 |
| PIV | FEF50_Base | 0.528647 | No | 2.368701 |
| PIV | FEF75_Base | 0.43698 | No | 3.392084 |
| PIV | MMEF_Base | 0.72746 | No | 1.401859 |
| AdV | FVC_Base | 0.12136 | No | -3.18481 |
| AdV | FEV1_Base | 0.44824 | No | -1.77892 |
| AdV | FEV1/FVC_Base | 0.128021 | No | 1.850141 |
| AdV | FEF25_Base | 0.698041 | No | 1.028351 |
| AdV | FEF50_Base | 0.702966 | No | 1.224336 |
| AdV | FEF75_Base | 0.661509 | No | -1.6153 |
| AdV | MMEF_Base | 0.712494 | No | -1.24852 |
| hMPV | FVC_Base | 0.758029 | No | -0.9147 |
| hMPV | FEV1_Base | 0.352419 | No | 3.115099 |
| hMPV | FEV1/FVC_Base | 0.000457 | Yes | 5.471945 |
| hMPV | FEF25_Base | 0.208314 | No | 4.66343 |
| hMPV | FEF50_Base | 0.068981 | No | 8.336672 |
| hMPV | FEF75_Base | 0.112602 | No | 8.564713 |
| hMPV | MMEF_Base | 0.161022 | No | 7.009358 |
| RSV | FVC_Base | 0.96856 | No | -0.09136 |
| RSV | FEV1_Base | 0.537327 | No | 1.582253 |
| RSV | FEV1/FVC_Base | 0.06915 | No | 2.404861 |
| RSV | FEF25_Base | 0.819109 | No | 0.652143 |
| RSV | FEF50_Base | 0.472428 | No | 2.546628 |
| RSV | FEF75_Base | 0.648571 | No | 1.868938 |
| RSV | MMEF_Base | 0.703777 | No | 1.419942 |
| CPn | FVC_Base | 0.875791 | No | -0.30767 |
| CPn | FEV1_Base | 0.32561 | No | 2.216434 |
| CPn | FEV1/FVC_Base | 0.01379 | Yes | 3.29902 |
| CPn | FEF25_Base | 0.648488 | No | 1.114847 |
| CPn | FEF50_Base | 0.161079 | No | 4.390377 |
| CPn | FEF75_Base | 0.106217 | No | 6.145745 |
| CPn | MMEF_Base | 0.197522 | No | 4.313656 |
| LP | FVC_Base | 0.494361 | No | -2.00411 |
| LP | FEV1_Base | 0.716976 | No | 1.169178 |
| LP | FEV1/FVC_Base | 0.004736 | Yes | 4.581764 |
| LP | FEF25_Base | 0.665785 | No | 1.548958 |
| LP | FEF50_Base | 0.246451 | No | 5.149021 |
| LP | FEF75_Base | 0.370806 | No | 4.747497 |
| LP | MMEF_Base | 0.494397 | No | 3.328891 |
| No_positive_result | FVC_Base | 0.058814 | No | 3.422341 |
| No_positive_result | FEV1_Base | 0.023597 | Yes | 4.072769 |
| No_positive_result | FEV1/FVC_Base | 0.284981 | No | 1.003578 |
| No_positive_result | FEF25_Base | 0.128211 | No | 3.11472 |
| No_positive_result | FEF50_Base | 0.041745 | Yes | 4.613136 |
| No_positive_result | FEF75_Base | 0.035369 | Yes | 6.129357 |
| No_positive_result | MMEF_Base | 0.025467 | Yes | 5.483701 |
| RhV | FVC_Base | 0.27547 | No | -3.55905 |
| RhV | FEV1_Base | 0.176344 | No | -4.72356 |
| RhV | FEV1/FVC_Base | 0.197021 | No | -2.22481 |
| RhV | FEF25_Base | 0.135919 | No | -6.01851 |
| RhV | FEF50_Base | 0.028074 | Yes | -9.7671 |
| RhV | FEF75_Base | 0.005003 | Yes | -13.0608 |
| RhV | MMEF_Base | 0.043022 | Yes | -9.08385 |
| Flu_A | FVC_Base | 0.157333 | No | -2.83431 |
| Flu_A | FEV1_Base | 0.763384 | No | -0.70242 |
| Flu_A | FEV1/FVC_Base | 0.043154 | Yes | 2.723655 |
| Flu_A | FEF25_Base | 0.62948 | No | -1.23459 |
| Flu_A | FEF50_Base | 0.385106 | No | 2.894949 |
| Flu_A | FEF75_Base | 0.288939 | No | 4.291705 |
| Flu_A | MMEF_Base | 0.617805 | No | 1.75248 |
| Flu_B | FVC_Base | 0.282507 | No | -2.24682 |
| Flu_B | FEV1_Base | 0.93567 | No | -0.19782 |
| Flu_B | FEV1/FVC_Base | 0.050314 | No | 2.820518 |
| Flu_B | FEF25_Base | 0.938337 | No | 0.211065 |
| Flu_B | FEF50_Base | 0.418598 | No | 2.832615 |
| Flu_B | FEF75_Base | 0.359055 | No | 4.102415 |
| Flu_B | MMEF_Base | 0.674733 | No | 1.622827 |

Table 6. Impact of pathogens on lung function using t-test.

| Condition | LungFunction | PValue | Significant | MeanDifference |
| --- | --- | --- | --- | --- |
| No_Pulmonary_Complications | FVC_Base | 0.093994 | No | 2.313693 |
| No_Pulmonary_Complications | FEV1_Base | 0.028067 | Yes | 3.23231 |
| No_Pulmonary_Complications | FEV1/FVC_Base | 0.183859 | No | 1.003042 |
| No_Pulmonary_Complications | FEF25_Base | 0.88475 | No | 0.240541 |
| No_Pulmonary_Complications | FEF50_Base | 0.226636 | No | 2.317687 |
| No_Pulmonary_Complications | FEF75_Base | 0.011885 | Yes | 5.9007 |
| No_Pulmonary_Complications | MMEF_Base | 0.089617 | No | 3.44309 |
| Lung_Consolidation | FVC_Base | 0.024092 | Yes | -2.90827 |
| Lung_Consolidation | FEV1_Base | 0.013387 | Yes | -3.37516 |
| Lung_Consolidation | FEV1/FVC_Base | 0.516842 | No | -0.46954 |
| Lung_Consolidation | FEF25_Base | 0.603172 | No | -0.81193 |
| Lung_Consolidation | FEF50_Base | 0.137374 | No | -2.70528 |
| Lung_Consolidation | FEF75_Base | 0.075995 | No | -3.91559 |
| Lung_Consolidation | MMEF_Base | 0.151644 | No | -2.76349 |
| Severe_Pneumonia | FVC_Base | 0.175826 | No | 2.019016 |
| Severe_Pneumonia | FEV1_Base | 0.785229 | No | 0.409037 |
| Severe_Pneumonia | FEV1/FVC_Base | 0.006257 | Yes | -2.25916 |
| Severe_Pneumonia | FEF25_Base | 0.501953 | No | 1.225323 |
| Severe_Pneumonia | FEF50_Base | 0.870167 | No | -0.33946 |
| Severe_Pneumonia | FEF75_Base | 0.025282 | Yes | -5.56228 |
| Severe_Pneumonia | MMEF_Base | 0.266378 | No | -2.38164 |
| Pleural_Effusion | FVC_Base | 0.897406 | No | 0.269784 |
| Pleural_Effusion | FEV1_Base | 0.928342 | No | -0.17862 |
| Pleural_Effusion | FEV1/FVC_Base | 0.626179 | No | -0.56222 |
| Pleural_Effusion | FEF25_Base | 0.574774 | No | 1.412739 |
| Pleural_Effusion | FEF50_Base | 0.595499 | No | -1.50076 |
| Pleural_Effusion | FEF75_Base | 0.333439 | No | -3.29676 |
| Pleural_Effusion | MMEF_Base | 0.582079 | No | -1.60772 |
| Hypoxemia | FVC_Base | 0.122608 | No | -4.36335 |
| Hypoxemia | FEV1_Base | 0.296731 | No | -2.79089 |
| Hypoxemia | FEV1/FVC_Base | 0.432517 | No | 2.051088 |
| Hypoxemia | FEF25_Base | 0.123356 | No | 5.158187 |
| Hypoxemia | FEF50_Base | 0.578378 | No | -2.55634 |
| Hypoxemia | FEF75_Base | 0.393771 | No | -6.08696 |
| Hypoxemia | MMEF_Base | 0.548249 | No | -3.34225 |
| Atelectasis | FVC_Base | 0.037986 | Yes | -6.72626 |
| Atelectasis | FEV1_Base | 0.098188 | No | -5.27629 |
| Atelectasis | FEV1/FVC_Base | 0.163969 | No | 2.163573 |
| Atelectasis | FEF25_Base | 0.488337 | No | 2.534342 |
| Atelectasis | FEF50_Base | 0.595393 | No | 2.521713 |
| Atelectasis | FEF75_Base | 0.560794 | No | 3.085351 |
| Atelectasis | MMEF_Base | 0.589705 | No | 2.622546 |
| No_Pulmonary_Complications | FVC_1month | 0.009317 | Yes | 4.018177 |
| No_Pulmonary_Complications | FEV1_1month | 0.001851 | Yes | 5.288345 |
| No_Pulmonary_Complications | FEV1/FVC_1month | 0.297498 | No | 1.039296 |
| No_Pulmonary_Complications | FEF25_1month | 0.232841 | No | 2.753863 |
| No_Pulmonary_Complications | FEF50_1month | 0.081808 | No | 4.744821 |
| No_Pulmonary_Complications | FEF75_1month | 0.0015 | Yes | 9.953276 |
| No_Pulmonary_Complications | MMEF_1month | 0.007453 | Yes | 7.161617 |
| Lung_Consolidation | FVC_1month | 0.124383 | No | -2.31478 |
| Lung_Consolidation | FEV1_1month | 0.03962 | Yes | -3.50897 |
| Lung_Consolidation | FEV1/FVC_1month | 0.298285 | No | -0.99779 |
| Lung_Consolidation | FEF25_1month | 0.333774 | No | -2.2285 |
| Lung_Consolidation | FEF50_1month | 0.201641 | No | -3.43052 |
| Lung_Consolidation | FEF75_1month | 0.002945 | Yes | -9.09408 |
| Lung_Consolidation | MMEF_1month | 0.027402 | Yes | -5.89988 |
| Severe_Pneumonia | FVC_1month | 0.831412 | No | -0.3408 |
| Severe_Pneumonia | FEV1_1month | 0.061294 | No | -3.55677 |
| Severe_Pneumonia | FEV1/FVC_1month | 0.001436 | Yes | -3.29307 |
| Severe_Pneumonia | FEF25_1month | 0.343011 | No | -2.43199 |
| Severe_Pneumonia | FEF50_1month | 0.134466 | No | -4.37963 |
| Severe_Pneumonia | FEF75_1month | 0.005589 | Yes | -9.33834 |
| Severe_Pneumonia | MMEF_1month | 0.021205 | Yes | -6.85543 |
| Pleural_Effusion | FVC_1month | 0.93103 | No | -0.24712 |
| Pleural_Effusion | FEV1_1month | 0.500493 | No | -2.19226 |
| Pleural_Effusion | FEV1/FVC_1month | 0.133095 | No | -2.61891 |
| Pleural_Effusion | FEF25_1month | 0.638003 | No | -2.26307 |
| Pleural_Effusion | FEF50_1month | 0.234546 | No | -5.8909 |
| Pleural_Effusion | FEF75_1month | 0.070888 | No | -9.05757 |
| Pleural_Effusion | MMEF_1month | 0.162819 | No | -6.75676 |
| Hypoxemia | FVC_1month | 0.11279 | No | -6.87335 |
| Hypoxemia | FEV1_1month | 0.267087 | No | -4.53671 |
| Hypoxemia | FEV1/FVC_1month | 0.146631 | No | 2.467484 |
| Hypoxemia | FEF25_1month | 0.129653 | No | 3.805065 |
| Hypoxemia | FEF50_1month | 0.315943 | No | -4.3749 |
| Hypoxemia | FEF75_1month | 0.331659 | No | -4.95226 |
| Hypoxemia | MMEF_1month | 0.296199 | No | -3.13706 |
| Atelectasis | FVC_1month | 0.030802 | Yes | -8.97928 |
| Atelectasis | FEV1_1month | 0.075171 | No | -6.6415 |
| Atelectasis | FEV1/FVC_1month | 0.204156 | No | 2.934036 |
| Atelectasis | FEF25_1month | 0.821793 | No | -1.42595 |
| Atelectasis | FEF50_1month | 0.778811 | No | -1.46105 |
| Atelectasis | FEF75_1month | 0.828813 | No | 1.112149 |
| Atelectasis | MMEF_1month | 0.882809 | No | -0.72911 |

Table 7. Impact of pulmonary complications on lung function using t-test.

| Independent_Var | Dependent_Var | Effect_Size | P_Value |
| --- | --- | --- | --- |
| Age | FEV1_FVC_Base | 0.043928 | 0.001118 |
| Sex | FEV1_FVC_Base | -2.50693 | 0.000473 |
| MP | FEV1_FVC_Base | -0.74252 | 0.350843 |
| PIV | FEV1_FVC_Base | 2.811614 | 0.036422 |
| AdV | FEV1_FVC_Base | 1.842417 | 0.117993 |
| hMPV | FEV1_FVC_Base | 5.456719 | 0.000536 |
| RSV | FEV1_FVC_Base | 2.395079 | 0.054605 |
| CPn | FEV1_FVC_Base | 3.280975 | 0.006407 |
| LP | FEV1_FVC_Base | 4.570765 | 0.004364 |
| No_positive_result | FEV1_FVC_Base | 0.996989 | 0.297139 |
| RhV | FEV1_FVC_Base | -2.21075 | 0.207372 |
| Flu_A | FEV1_FVC_Base | 2.709225 | 0.022359 |
| Flu_B | FEV1_FVC_Base | 2.808609 | 0.026721 |
| EBV | FEV1_FVC_Base | 1.225078 | 0.70724 |
| No_Pulmonary_Complications | FEV1_FVC_Base | 1.003042 | 0.179033 |
| Lung_Consolidation | FEV1_FVC_Base | -0.46954 | 0.515617 |
| Severe_Pneumonia | FEV1_FVC_Base | -2.25916 | 0.007384 |
| Pleural_Effusion | FEV1_FVC_Base | -0.56222 | 0.636234 |
| Hypoxemia | FEV1_FVC_Base | 2.051088 | 0.432118 |
| Atelectasis | FEV1_FVC_Base | 2.163573 | 0.226264 |
| Age | FVC_Base | -0.03135 | 0.197516 |
| Sex | FVC_Base | -0.59533 | 0.646053 |
| MP | FVC_Base | -3.6309 | 0.010967 |
| PIV | FVC_Base | -1.56129 | 0.518896 |
| AdV | FVC_Base | -3.38004 | 0.110672 |
| hMPV | FVC_Base | -1.12057 | 0.694051 |
| RSV | FVC_Base | -0.30885 | 0.890536 |
| CPn | FVC_Base | -0.52515 | 0.808854 |
| LP | FVC_Base | -2.20545 | 0.445783 |
| No_positive_result | FVC_Base | 3.133765 | 0.068074 |
| RhV | FVC_Base | -3.7548 | 0.23367 |
| Flu_A | FVC_Base | -3.03239 | 0.155608 |
| Flu_B | FVC_Base | -2.44888 | 0.283418 |
| EBV | FVC_Base | 3.4209 | 0.559658 |
| No_Pulmonary_Complications | FVC_Base | 2.313693 | 0.084553 |
| Lung_Consolidation | FVC_Base | -2.90827 | 0.024816 |
| Severe_Pneumonia | FVC_Base | 2.019016 | 0.184022 |
| Pleural_Effusion | FVC_Base | 0.269784 | 0.899557 |
| Hypoxemia | FVC_Base | -4.36335 | 0.352577 |
| Atelectasis | FVC_Base | -6.72626 | 0.036168 |
| Age | FEV1_Base | -0.00218 | 0.932607 |
| Sex | FEV1_Base | -1.04325 | 0.447444 |
| MP | FEV1_Base | -4.43921 | 0.003302 |
| PIV | FEV1_Base | 0.347153 | 0.892341 |
| AdV | FEV1_Base | -2.04402 | 0.362951 |
| hMPV | FEV1_Base | 2.837528 | 0.347005 |
| RSV | FEV1_Base | 1.295286 | 0.585874 |
| CPn | FEV1_Base | 1.921478 | 0.403379 |
| LP | FEV1_Base | 0.900149 | 0.769028 |
| No_positive_result | FEV1_Base | 3.711622 | 0.041323 |
| RhV | FEV1_Base | -4.9703 | 0.136637 |
| Flu_A | FEV1_Base | -0.97583 | 0.666518 |
| Flu_B | FEV1_Base | -0.47119 | 0.84563 |
| EBV | FEV1_Base | 3.892001 | 0.53105 |
| No_Pulmonary_Complications | FEV1_Base | 3.23231 | 0.022816 |
| Lung_Consolidation | FEV1_Base | -3.37516 | 0.013921 |
| Severe_Pneumonia | FEV1_Base | 0.409037 | 0.799632 |
| Pleural_Effusion | FEV1_Base | -0.17862 | 0.937132 |
| Hypoxemia | FEV1_Base | -2.79089 | 0.574767 |
| Atelectasis | FEV1_Base | -5.27629 | 0.121214 |
| Age | FEF25_Base | 0.031642 | 0.281877 |
| Sex | FEF25_Base | 1.348499 | 0.389157 |
| MP | FEF25_Base | -4.33049 | 0.012049 |
| PIV | FEF25_Base | -0.0875 | 0.976136 |
| AdV | FEF25_Base | 0.823607 | 0.747949 |
| hMPV | FEF25_Base | 4.458434 | 0.194915 |
| RSV | FEF25_Base | 0.45383 | 0.867078 |
| CPn | FEF25_Base | 0.911067 | 0.728337 |
| LP | FEF25_Base | 1.357146 | 0.697839 |
| No_positive_result | FEF25_Base | 2.857378 | 0.168835 |
| RhV | FEF25_Base | -6.18309 | 0.104378 |
| Flu_A | FEF25_Base | -1.42072 | 0.582189 |
| Flu_B | FEF25_Base | 0.016686 | 0.995176 |
| EBV | FEF25_Base | 1.431487 | 0.839917 |
| No_Pulmonary_Complications | FEF25_Base | 0.240541 | 0.882171 |
| Lung_Consolidation | FEF25_Base | -0.81193 | 0.604839 |
| Severe_Pneumonia | FEF25_Base | 1.225323 | 0.504854 |
| Pleural_Effusion | FEF25_Base | 1.412739 | 0.584311 |
| Hypoxemia | FEF25_Base | 5.158187 | 0.363089 |
| Atelectasis | FEF25_Base | 2.534342 | 0.514299 |
| Age | FEF50_Base | 0.101977 | 0.002811 |
| Sex | FEF50_Base | -1.54627 | 0.396585 |
| MP | FEF50_Base | -4.65698 | 0.0205 |
| PIV | FEF50_Base | 2.223467 | 0.513936 |
| AdV | FEF50_Base | 1.079218 | 0.71772 |
| hMPV | FEF50_Base | 8.174807 | 0.041088 |
| RSV | FEF50_Base | 2.395296 | 0.448098 |
| CPn | FEF50_Base | 4.222301 | 0.166637 |
| LP | FEF50_Base | 5.001024 | 0.219077 |
| No_positive_result | FEF50_Base | 4.4003 | 0.068711 |
| RhV | FEF50_Base | -9.86219 | 0.026006 |
| Flu_A | FEF50_Base | 2.736945 | 0.36273 |
| Flu_B | FEF50_Base | 2.680622 | 0.404208 |
| EBV | FEF50_Base | 3.388237 | 0.681438 |
| No_Pulmonary_Complications | FEF50_Base | 2.317687 | 0.219864 |
| Lung_Consolidation | FEF50_Base | -2.70528 | 0.138484 |
| Severe_Pneumonia | FEF50_Base | -0.33946 | 0.873999 |
| Pleural_Effusion | FEF50_Base | -1.50076 | 0.617814 |
| Hypoxemia | FEF50_Base | -2.55634 | 0.698867 |
| Atelectasis | FEF50_Base | 2.521713 | 0.577476 |
| Age | FEF75_Base | 0.164801 | 6.49E-05 |
| Sex | FEF75_Base | -5.15183 | 0.019585 |
| MP | FEF75_Base | -3.93649 | 0.106691 |
| PIV | FEF75_Base | 3.459111 | 0.40229 |
| AdV | FEF75_Base | -1.51255 | 0.676059 |
| hMPV | FEF75_Base | 8.614913 | 0.075992 |
| RSV | FEF75_Base | 1.944094 | 0.611675 |
| CPn | FEF75_Base | 6.187618 | 0.094487 |
| LP | FEF75_Base | 4.813664 | 0.329403 |
| No_positive_result | FEF75_Base | 6.13755 | 0.036183 |
| RhV | FEF75_Base | -12.9346 | 0.01601 |
| Flu_A | FEF75_Base | 4.346857 | 0.233086 |
| Flu_B | FEF75_Base | 4.162648 | 0.28533 |
| EBV | FEF75_Base | -7.84608 | 0.432983 |
| No_Pulmonary_Complications | FEF75_Base | 5.9007 | 0.009826 |
| Lung_Consolidation | FEF75_Base | -3.91559 | 0.076882 |
| Severe_Pneumonia | FEF75_Base | -5.56228 | 0.03177 |
| Pleural_Effusion | FEF75_Base | -3.29676 | 0.365923 |
| Hypoxemia | FEF75_Base | -6.08696 | 0.447376 |
| Atelectasis | FEF75_Base | 3.085351 | 0.574047 |
| Age | MMEF_Base | 0.098765 | 0.006203 |
| Sex | MMEF_Base | -2.44009 | 0.205294 |
| MP | MMEF_Base | -4.91282 | 0.0207 |
| PIV | MMEF_Base | 1.320841 | 0.713672 |
| AdV | MMEF_Base | -1.31326 | 0.677153 |
| hMPV | MMEF_Base | 6.909965 | 0.102464 |
| RSV | MMEF_Base | 1.336009 | 0.68888 |
| CPn | MMEF_Base | 4.206062 | 0.192286 |
| LP | MMEF_Base | 3.245006 | 0.450597 |
| No_positive_result | MMEF_Base | 5.323399 | 0.037053 |
| RhV | MMEF_Base | -9.12469 | 0.051375 |
| Flu_A | MMEF_Base | 1.663727 | 0.600619 |
| Flu_B | MMEF_Base | 1.538229 | 0.650611 |
| EBV | MMEF_Base | -4.00606 | 0.645972 |
| No_Pulmonary_Complications | MMEF_Base | 3.44309 | 0.084324 |
| Lung_Consolidation | MMEF_Base | -2.76349 | 0.152091 |
| Severe_Pneumonia | MMEF_Base | -2.38164 | 0.292105 |
| Pleural_Effusion | MMEF_Base | -1.60772 | 0.61294 |
| Hypoxemia | MMEF_Base | -3.34225 | 0.632147 |
| Atelectasis | MMEF_Base | 2.622546 | 0.583475 |
| Age | LAD_Base | 0.991853 | 0.041849 |
| Sex | LAD_Base | 2.155669 | 0.000467 |
| MP | LAD_Base | 1.429507 | 0.146437 |
| PIV | LAD_Base | 0.619662 | 0.290296 |
| AdV | LAD_Base | 0.921205 | 0.815783 |
| hMPV | LAD_Base | 0.266458 | 0.07346 |
| RSV | LAD_Base | 0.605926 | 0.233784 |
| CPn | LAD_Base | 0.457358 | 0.079161 |
| LP | LAD_Base | 0.43527 | 0.178267 |
| No_positive_result | LAD_Base | 0.836579 | 0.539382 |
| RhV | LAD_Base | 1.295929 | 0.589666 |
| Flu_A | LAD_Base | 0.829248 | 0.607801 |
| Flu_B | LAD_Base | 0.637209 | 0.285543 |
| EBV | LAD_Base | 0.672673 | 0.714866 |
| No_Pulmonary_Complications | LAD_Base | 0.892506 | 0.606486 |
| Lung_Consolidation | LAD_Base | 1.214876 | 0.356664 |
| Severe_Pneumonia | LAD_Base | 1.441549 | 0.120924 |
| Pleural_Effusion | LAD_Base | 1.19349 | 0.59662 |
| Hypoxemia | LAD_Base | 0.89899 | 0.892637 |
| Atelectasis | LAD_Base | 0.803704 | 0.695431 |
| Age | SAD_Base | 0.9945 | 0.090146 |
| Sex | SAD_Base | 1.178196 | 0.338506 |
| MP | SAD_Base | 1.754902 | 0.004195 |
| PIV | SAD_Base | 0.913406 | 0.778602 |
| AdV | SAD_Base | 0.85473 | 0.580409 |
| hMPV | SAD_Base | 0.581242 | 0.180668 |
| RSV | SAD_Base | 0.754162 | 0.35485 |
| CPn | SAD_Base | 0.553271 | 0.055511 |
| LP | SAD_Base | 0.718182 | 0.404524 |
| No_positive_result | SAD_Base | 0.602239 | 0.033632 |
| RhV | SAD_Base | 2.716511 | 0.018938 |
| Flu_A | SAD_Base | 0.811975 | 0.469095 |
| Flu_B | SAD_Base | 0.732821 | 0.318539 |
| EBV | SAD_Base | 0.580702 | 0.518138 |
| No_Pulmonary_Complications | SAD_Base | 0.753247 | 0.113726 |
| Lung_Consolidation | SAD_Base | 1.419676 | 0.041449 |
| Severe_Pneumonia | SAD_Base | 1.005714 | 0.977357 |
| Pleural_Effusion | SAD_Base | 1.308597 | 0.333986 |
| Hypoxemia | SAD_Base | 1.773214 | 0.349053 |
| Atelectasis | SAD_Base | 1.045455 | 0.916342 |
| Age | FVC_1month | -0.05672 | 0.044646 |
| Sex | FVC_1month | 0.398845 | 0.791774 |
| MP | FVC_1month | -0.90951 | 0.565086 |
| PIV | FVC_1month | 0.595591 | 0.803366 |
| AdV | FVC_1month | -1.25561 | 0.555234 |
| hMPV | FVC_1month | -0.71707 | 0.776295 |
| RSV | FVC_1month | 2.376805 | 0.272645 |
| CPn | FVC_1month | 0.466741 | 0.839747 |
| LP | FVC_1month | 0.6788 | 0.787944 |
| No_positive_result | FVC_1month | 3.598881 | 0.047058 |
| RhV | FVC_1month | 5.492667 | 0.360745 |
| Flu_A | FVC_1month | -0.01827 | 0.993684 |
| Flu_B | FVC_1month | -1.11509 | 0.641048 |
| EBV | FVC_1month | -12.2891 | 0.193458 |
| No_Pulmonary_Complications | FVC_1month | 4.018177 | 0.008701 |
| Lung_Consolidation | FVC_1month | -2.31478 | 0.123966 |
| Severe_Pneumonia | FVC_1month | -0.3408 | 0.83575 |
| Pleural_Effusion | FVC_1month | -0.24712 | 0.925465 |
| Hypoxemia | FVC_1month | -6.87335 | 0.30547 |
| Atelectasis | FVC_1month | -8.97928 | 0.045873 |
| Age | FEV1_1month | -0.02402 | 0.452391 |
| Sex | FEV1_1month | -0.73698 | 0.665499 |
| MP | FEV1_1month | -0.27751 | 0.876417 |
| PIV | FEV1_1month | 6.922724 | 0.009931 |
| AdV | FEV1_1month | 3.511609 | 0.143144 |
| hMPV | FEV1_1month | 5.891431 | 0.03791 |
| RSV | FEV1_1month | 7.131808 | 0.003343 |
| CPn | FEV1_1month | 6.128543 | 0.018106 |
| LP | FEV1_1month | 6.567892 | 0.020551 |
| No_positive_result | FEV1_1month | 4.584246 | 0.024865 |
| RhV | FEV1_1month | -4.94567 | 0.465958 |
| Flu_A | FEV1_1month | 5.631556 | 0.029988 |
| Flu_B | FEV1_1month | 4.102673 | 0.127841 |
| EBV | FEV1_1month | -6.72173 | 0.528916 |
| No_Pulmonary_Complications | FEV1_1month | 5.288345 | 0.002169 |
| Lung_Consolidation | FEV1_1month | -3.50897 | 0.038483 |
| Severe_Pneumonia | FEV1_1month | -3.55677 | 0.05446 |
| Pleural_Effusion | FEV1_1month | -2.19226 | 0.461874 |
| Hypoxemia | FEV1_1month | -4.53671 | 0.549131 |
| Atelectasis | FEV1_1month | -6.6415 | 0.191466 |
| Age | FEV1_FVC_1month | 0.039822 | 0.026757 |
| Sex | FEV1_FVC_1month | -3.00816 | 0.001625 |
| MP | FEV1_FVC_1month | 1.057376 | 0.29331 |
| PIV | FEV1_FVC_1month | 6.773164 | 6.21E-06 |
| AdV | FEV1_FVC_1month | 5.298071 | 7.59E-05 |
| hMPV | FEV1_FVC_1month | 7.057845 | 8.13E-06 |
| RSV | FEV1_FVC_1month | 5.132532 | 0.000171 |
| CPn | FEV1_FVC_1month | 6.17102 | 2.05E-05 |
| LP | FEV1_FVC_1month | 6.309799 | 7.01E-05 |
| No_positive_result | FEV1_FVC_1month | 0.824935 | 0.475981 |
| RhV | FEV1_FVC_1month | -10.9595 | 0.003968 |
| Flu_A | FEV1_FVC_1month | 5.883606 | 5E-05 |
| Flu_B | FEV1_FVC_1month | 5.457998 | 0.000294 |
| EBV | FEV1_FVC_1month | 8.263686 | 0.169649 |
| No_Pulmonary_Complications | FEV1_FVC_1month | 1.039296 | 0.28885 |
| Lung_Consolidation | FEV1_FVC_1month | -0.99779 | 0.29826 |
| Severe_Pneumonia | FEV1_FVC_1month | -3.29307 | 0.001518 |
| Pleural_Effusion | FEV1_FVC_1month | -2.61891 | 0.118796 |
| Hypoxemia | FEV1_FVC_1month | 2.467484 | 0.563708 |
| Atelectasis | FEV1_FVC_1month | 2.934036 | 0.306779 |
| Age | FEF25_1month | 0.027013 | 0.53054 |
| Sex | FEF25_1month | 0.573623 | 0.802763 |
| MP | FEF25_1month | 0.409014 | 0.864893 |
| PIV | FEF25_1month | 4.510015 | 0.214321 |
| AdV | FEF25_1month | 5.935977 | 0.065899 |
| hMPV | FEF25_1month | 4.84008 | 0.206516 |
| RSV | FEF25_1month | 3.253391 | 0.323356 |
| CPn | FEF25_1month | 1.323318 | 0.706039 |
| LP | FEF25_1month | 4.342576 | 0.257182 |
| No_positive_result | FEF25_1month | 4.948771 | 0.072618 |
| RhV | FEF25_1month | -17.7165 | 0.051921 |
| Flu_A | FEF25_1month | 2.368188 | 0.499569 |
| Flu_B | FEF25_1month | 1.80894 | 0.618801 |
| EBV | FEF25_1month | 9.039167 | 0.529635 |
| No_Pulmonary_Complications | FEF25_1month | 2.753863 | 0.23895 |
| Lung_Consolidation | FEF25_1month | -2.2285 | 0.330492 |
| Severe_Pneumonia | FEF25_1month | -2.43199 | 0.330058 |
| Pleural_Effusion | FEF25_1month | -2.26307 | 0.572947 |
| Hypoxemia | FEF25_1month | 3.805065 | 0.70921 |
| Atelectasis | FEF25_1month | -1.42595 | 0.835298 |
| Age | FEF50_1month | 0.056674 | 0.260148 |
| Sex | FEF50_1month | -0.49818 | 0.85284 |
| MP | FEF50_1month | 1.892709 | 0.500606 |
| PIV | FEF50_1month | 10.36148 | 0.014351 |
| AdV | FEF50_1month | 9.363407 | 0.012906 |
| hMPV | FEF50_1month | 10.1006 | 0.023782 |
| RSV | FEF50_1month | 7.058562 | 0.066386 |
| CPn | FEF50_1month | 6.651587 | 0.104269 |
| LP | FEF50_1month | 9.044749 | 0.043108 |
| No_positive_result | FEF50_1month | 5.367573 | 0.096016 |
| RhV | FEF50_1month | -20.2476 | 0.057508 |
| Flu_A | FEF50_1month | 7.09169 | 0.083187 |
| Flu_B | FEF50_1month | 6.161956 | 0.14664 |
| EBV | FEF50_1month | 19.00186 | 0.258165 |
| No_Pulmonary_Complications | FEF50_1month | 4.744821 | 0.082325 |
| Lung_Consolidation | FEF50_1month | -3.43052 | 0.199938 |
| Severe_Pneumonia | FEF50_1month | -4.37963 | 0.133225 |
| Pleural_Effusion | FEF50_1month | -5.8909 | 0.209082 |
| Hypoxemia | FEF50_1month | -4.3749 | 0.713878 |
| Atelectasis | FEF50_1month | -1.46105 | 0.85545 |
| Age | FEF75_1month | 0.151956 | 0.008434 |
| Sex | FEF75_1month | -3.98848 | 0.196515 |
| MP | FEF75_1month | 1.552712 | 0.631357 |
| PIV | FEF75_1month | 15.44288 | 0.001474 |
| AdV | FEF75_1month | 12.94532 | 0.00277 |
| hMPV | FEF75_1month | 15.28699 | 0.002877 |
| RSV | FEF75_1month | 11.9779 | 0.00664 |
| CPn | FEF75_1month | 12.72291 | 0.00675 |
| LP | FEF75_1month | 13.12159 | 0.010663 |
| No_positive_result | FEF75_1month | 6.591532 | 0.075757 |
| RhV | FEF75_1month | -25.2767 | 0.039349 |
| Flu_A | FEF75_1month | 13.74982 | 0.003384 |
| Flu_B | FEF75_1month | 10.60024 | 0.029761 |
| EBV | FEF75_1month | 27.71234 | 0.151811 |
| No_Pulmonary_Complications | FEF75_1month | 9.953276 | 0.001456 |
| Lung_Consolidation | FEF75_1month | -9.09408 | 0.002997 |
| Severe_Pneumonia | FEF75_1month | -9.33834 | 0.005222 |
| Pleural_Effusion | FEF75_1month | -9.05757 | 0.093137 |
| Hypoxemia | FEF75_1month | -4.95226 | 0.718492 |
| Atelectasis | FEF75_1month | 1.112149 | 0.904138 |
| Age | MMEF_1month | 0.069402 | 0.166493 |
| Sex | MMEF_1month | -1.40732 | 0.599166 |
| MP | MMEF_1month | 0.893507 | 0.749898 |
| PIV | MMEF_1month | 10.51816 | 0.012672 |
| AdV | MMEF_1month | 9.22116 | 0.01408 |
| hMPV | MMEF_1month | 10.14656 | 0.022756 |
| RSV | MMEF_1month | 7.641923 | 0.046137 |
| CPn | MMEF_1month | 7.281463 | 0.074398 |
| LP | MMEF_1month | 9.105028 | 0.041146 |
| No_positive_result | MMEF_1month | 6.692351 | 0.037178 |
| RhV | MMEF_1month | -22.8063 | 0.031773 |
| Flu_A | MMEF_1month | 7.736535 | 0.057922 |
| Flu_B | MMEF_1month | 6.260753 | 0.139126 |
| EBV | MMEF_1month | 17.91702 | 0.285027 |
| No_Pulmonary_Complications | MMEF_1month | 7.161617 | 0.008339 |
| Lung_Consolidation | MMEF_1month | -5.89988 | 0.026664 |
| Severe_Pneumonia | MMEF_1month | -6.85543 | 0.018114 |
| Pleural_Effusion | MMEF_1month | -6.75676 | 0.148346 |
| Hypoxemia | MMEF_1month | -3.13706 | 0.792054 |
| Atelectasis | MMEF_1month | -0.72911 | 0.927362 |
| Age | LAD_1month | 0.991086 | 0.121524 |
| Sex | LAD_1month | 2.22963 | 0.015806 |
| MP | LAD_1month | 1.445478 | 0.278624 |
| PIV | LAD_1month | 3.96E-08 | 0.987666 |
| AdV | LAD_1month | 0.208333 | 0.034084 |
| hMPV | LAD_1month | 1.1E-07 | 0.982005 |
| RSV | LAD_1month | 0.220238 | 0.041153 |
| CPn | LAD_1month | 3.91E-08 | 0.987138 |
| LP | LAD_1month | 1.1E-07 | 0.982005 |
| No_positive_result | LAD_1month | 0.347222 | 0.031832 |
| RhV | LAD_1month | 22.86957 | 0.005585 |
| Flu_A | LAD_1month | 0.125207 | 0.04277 |
| Flu_B | LAD_1month | 0.291667 | 0.098279 |
| EBV | LAD_1month | 9.1E-07 | 0.989216 |
| No_Pulmonary_Complications | LAD_1month | 1.131905 | 0.692277 |
| Lung_Consolidation | LAD_1month | 1.24187 | 0.482985 |
| Severe_Pneumonia | LAD_1month | 1.251082 | 0.494389 |
| Pleural_Effusion | LAD_1month | 1.164251 | 0.769679 |
| Hypoxemia | LAD_1month | 9.03E-07 | 0.984741 |
| Atelectasis | LAD_1month | 3.26E-07 | 0.985101 |
| Age | SAD_1month | 0.995858 | 0.424907 |
| Sex | SAD_1month | 1.103189 | 0.724648 |
| MP | SAD_1month | 0.992485 | 0.979302 |
| PIV | SAD_1month | 7.59E-08 | 0.980441 |
| AdV | SAD_1month | 0.144886 | 0.008758 |
| hMPV | SAD_1month | 7.73E-08 | 0.981613 |
| RSV | SAD_1month | 0.153274 | 0.011007 |
| CPn | SAD_1month | 0.184028 | 0.022232 |
| LP | SAD_1month | 7.73E-08 | 0.981613 |
| No_positive_result | SAD_1month | 0.494737 | 0.069808 |
| RhV | SAD_1month | 5.857143 | 0.055669 |
| Flu_A | SAD_1month | 0.087734 | 0.017405 |
| Flu_B | SAD_1month | 0.203598 | 0.031951 |
| EBV | SAD_1month | 6.47E-07 | 0.988952 |
| No_Pulmonary_Complications | SAD_1month | 0.602007 | 0.087666 |
| Lung_Consolidation | SAD_1month | 1.675032 | 0.065331 |
| Severe_Pneumonia | SAD_1month | 1.333443 | 0.327774 |
| Pleural_Effusion | SAD_1month | 1.572414 | 0.30739 |
| Hypoxemia | SAD_1month | 6.42E-07 | 0.984367 |
| Atelectasis | SAD_1month | 2.31E-07 | 0.984759 |

Table 8. Univariate Regression using linear regression and logistic regression.
